# The first case of cholecytitis caused by *Aggregatibacter kilianii* in Korea

**DOI:** 10.1186/s12879-022-07471-7

**Published:** 2022-05-26

**Authors:** Dahae Yang, Chang-Ki Kim, Junja Park, Na Yeong Song, Yunna Lee, Woonhyoung Lee

**Affiliations:** 1 Department of Laboratory Medicine, Kosin Gospel University Hospital, 262 Gamchen-ro, Seo-gu, 49267 Busan, Korea; 2 Seoul Clinical Laboratories, Yongin, Korea; 3 Department of Psychiatry, Kosin Gospel University Hospital, 262 Gamchen-ro, Seo-gu, 49267 Busan, Korea

**Keywords:** Aggregatibacter, *A. kilianii*, HACEK, Cholecystitis

## Abstract

**Background:**

The bacterial genus *Aggregatibacter* was categorized in 2006 to accommodate the former *Actinobacillus actinomycetemcomitans*, *Haemophilus aphrophilus*, and *H. segnis* species. *Aggregatibacter kilianii* is a normal resident of the human upper respiratory tract but can also cause serious infections. *A*. *kilianii* is relatively newly identified and has been isolated from conjunctivitis, wounds, abdominal abscesses, and blood.

**Case presentation:**

An 80-year-old female patient with distal common bile duct cancer was admitted to our hospital with sudden loss of consciousness and general weakness, fever, and abdominal pain for 3 days. Two colonial morphologies were isolated from both the blood and bile cultures; one was identified as *Streptococcus constellatus* subsp. *pharyngis*, but the other was not recognized by Vitek2 and MALDI-TOF. The 16 S rRNA sequences showed 99.73% similarity with the sequence of *A*. *kilianii* strains.

**Conclusion and discussion:**

This article presents the first case of a clinical isolate of *A.* *kilianii* outside Europe. This case is also the first of the antimicrobial profile of this strain. This report highlights the importance of proper molecular identification for timely diagnosis and treatment of disease.

## Background

The HACEK group, comprised of bacterial genera *Haemophilus, Aggregatibacter, Cardiobacterium, Eikenella*, and *Kingella*, was created to describe a heterogeneous group of gram-negative bacteria that are commensal to the oropharyngeal and urogenital tract of humans [1]. The bacterial genus *Aggregatibacter* was categorized in 2006 to accommodate the former *Actinobacillus actinomycetemcomitans*, *Haemophilus aphrophilus*, and *H. segnis* species [2]. In 2018, a subspecies called *A. kilianii*, which is distinguished from the existing *Aggregatibacter* by genomic sequence and phenotype, was newly described [3]. *A. kilianii* is a normal resident of the human upper respiratory tract but can also cause serious infections. Here, we present the first reported case of cholecystitis caused by *A. kilianii*.

## Case presentation

An 80-year-old woman presented to the emergency department with loss of consciousness. Her daughter reported that she had been experiencing general weakness, fever, jaundice in the eye, and abdominal pain starting 3 days prior. She had been previously diagnosed with distal common bile duct cancer with para-aortic and hepato-duodenal lymph node metastasis and underwent endoscopic retrograde cholangiopancreatography and endoscopic retrograde biliary drainage (ERBD) a year ago. The patient had a temperature of 38.2 °C, a heart rate of 108 beats/min, a respiratory rate of 20 breaths/min, a blood pressure of 72/50 mmHg, and a SaO2 of 97%. Abdominal physical examination revealed tenderness without rebound on the upper right quadrant of the abdomen and a positive Murphy sign.

Biochemical analyses demonstrated leukocytosis and neutrophilia as follows: hemoglobin, 10.2 g/dL; white blood cell count, 24.79 × 10^9^/L (neutrophils: 83%, lymphocytes: 4%, monocytes: 7%, myelocytes: 1%), platelet count, 315 × 10^9^/L; and hypersensitive C-reactive protein (CRP), 20.797 mg/L (reference range 0-0.75 mg/dL). The hepatic enzymes showed mixed cholestatic and hepatic features: alkaline phosphatase, 904 U/L (reference range 25-100U/L); alanine aminotransferase, 105 U/L (reference range 3–40 U/L); and gamma-glutamyl-transferase, 204 U/L (reference range 0-50U/L). The total bilirubin level was 8.36 mg/dL (reference range 0.2–1.2 mg/dL), and the direct bilirubin was 7.68 mg/dL. Hepatic synthetic function values were as follows: albumin, 2.3 g/dL (reference range 3.5-5 g/dL); prothrombin time, 21.0 s (reference range 11–15 s); activated partial thromboplastin clotting time, 62.4 s (reference range 27–45 s); and D-dimer, 7.53 (reference range 0-0.5µg/mL).

Compared with previous computed tomography findings, gallbladder, common bile duct, and intrahepatic duct dilatation had deteriorated, suggesting biliary tract obstruction caused by ERBD malfunction followed by cholecystitis. ERBD repositioning or removal was needed but not applicable considering the general condition of the patient. Percutaneous transhepatic biliary drainage and percutaneous transhepatic gallbladder drainage were performed. Two sets of aerobic and anaerobic blood cultures and one set of bile cultures (BACT/ALERT Culture Media, bioMérieux, Marcy l’Etoile, France) were initiated prior to an intravenous infusion of 2 g cefotaxime every eight hours.

**Fig. 1:**
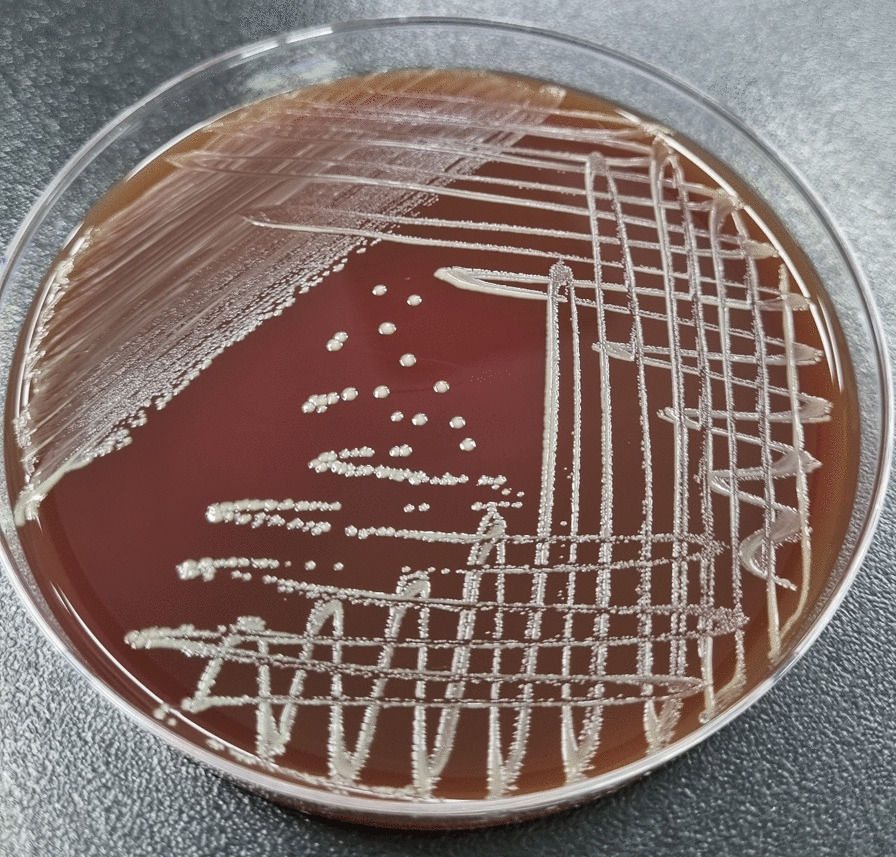
Isolated strain on blood agar that grew as granular, yellowish, opaque small colonies after 48–72 h incubation with 5% CO_2_

Bacterial growth was confirmed in the aerobic blood culture bottles after 21 h. The sample was cultured on MacConkey (MICROMEDIA Corp., Busan, Korea) and blood agars (MICROMEDIA Corp.) with 5% CO_2_ for 24 h. Two colonial morphologies were isolated from blood agar, but none grew on MacConkey agar. First one was identified as *Streptococcus constellatus* subsp. *pharyngis*. However, the other yellowish, opaque small colonies (Fig. 1) were poorly identified, with low discrimination quantity to *Aggregatibacter aphrophilus* by conventional biochemical identification methods using the Vitek2 NH card (bioMérieux). Similarly, many gram positive cocci and moderate gram negative bacilli in bile culture grew on blood agar after 24 h. Gram-positive cocci were identified as *Streptococcus constellatus* and other small interspersed colonies with few large colonies were poorly identified with *A. aphrophilus*.

The biochemical results were negative for urease, ornithine decarboxylase, α-arabinose, and N-acetyl‑D‑glucosamine and positive for D-galactose, phenylalanine arylamidase, and β-galactopyranosidase indoxyl.

Matrix-assisted laser desorption/ionization time of-flight (MALDI-TOF) mass spectrometry (Bruker Daltonics Inc., Billerica, MA, USA) also could not identify the isolate. The greatest similarities were obtained with *A.* *aphrophilus* strains, but identification was unreliable, with log scores in the range of 1.47 to 1.80. A single measurement exceeded the 1.7-log-score breakpoint, but the result was not reproducible.

**Fig. 2:**
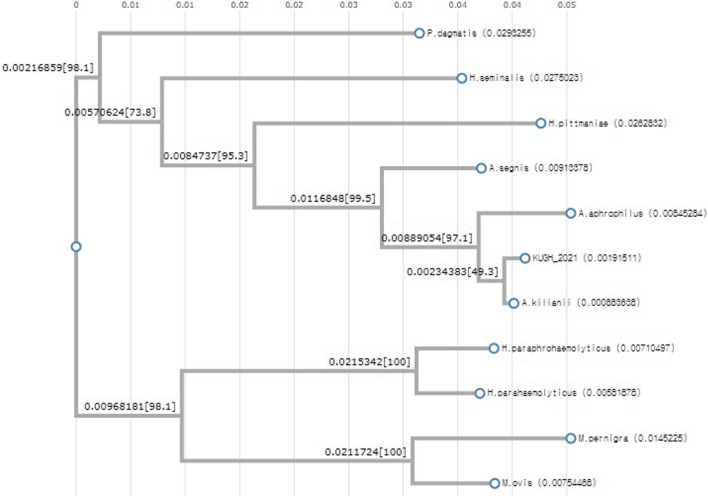
Phylogenetic tree of the isolate in this study (KUGH_2021) and 10 similar species based on 16 S rRNA gene sequences. The 16 S rRNA sequence (1474 bp) showed 99.73% similarity with that of *A*. *kiliani* strains. Alignment and phylogenetic reconstructions were performed using ETE tool kit v3.1.1 [9], as implemented on the GenomeNet. Branch supports are the Chi^2^-based parametric values returned by the approximate likelihood ratio test. GenBank accession numbers are as follows: *Aggregatibacter kilianii* (NRCN01000001.1), *Aggregatibacter aphrophilus* (LR134327.1), *Aggregatibacter segnis* (LS483443.1), *Haemophilus parahaemolyticus* (CP069510.1), *Haemophilus paraphrohaemolyticus* (UGHT01000002.1), *Haemophilus pittmaniae* (LT906463.1), *Haemophilus seminalis* (VXDF01000014.1), *Mannheimia ovis* (CP046531.1), *Mannheimia pernigra* (CP055302.1), *Pasteurella dagmatis* (LT906448.1)

We sequenced the 16 S rRNA gene for identification at the species level and obtained 1474 bp with 99.73% similarity with the sequence of *A.* *kilianii* strains (GenBank accession no: NRCN00000000.1, Fig. 2).

Antimicrobial susceptibility testing for ampicillin, amoxicillin-clavulanate, cefotaxime, ciprofloxacin, imipenem, and trimethoprim-sulfamethoxazole was performed by E-test method (bioMérieux, Marcy-l’Étoile, France) according to the manufacturer’s specifications. The minimum inhibitory concentrations (MICs) were determined visually as the lowest concentrations with no visible growth. The MICs values in our study were as follows; ampicillin (MIC 0.19 µg/mL), amoxicillin-clavulanate (MIC 0.38 µg/mL), cefotaxime (MIC 0.016 µg/mL), ciprofloxacin (MIC < 0.006 µg/mL), imipenem (MIC 1.0 µg/mL), and trimethoprim-sulfamethoxazole (MIC 0.023 µg/mL). The Clinical and Laboratory Standards Institute (CLSI) M45-A2 guideline provides only information on broth microdilution when interpreting the MICs of the HACEK group [4], so susceptibility interpretation was not performed.

In our case, because both bacteria responded to cefotaxime, initial treatment was continued. On day 3, CRP began to decrease, and the patient was able to make eye contact. On day 6, the patient was able to answer simple questions. On the 13th day, CRP decreased to 2.048 mg/L, and the patient’s general condition improved. Her family insisted on discharge, and she was released with oral antibiotics. A month later, follow-up was conducted by phone. The patient’s daughter said the infection had improved, but the patient’s general condition had worsened, and the family was planning to admit her to a hospice hospital.

## Discussion and conclusions

Conventional biochemical identification and MALDI-TOF are excellent methods for identifying bacteria. However, but it is difficult to adapt these existing processes to a new strain because their analyses rely on previously reported strain characteristics [5]. However, high-throughput sequencing can analyze a new strain and allow comparison of the PCR-amplified 16 S sequence with the standardized reference database for microbial classification for accurate identification [6]. A precise diagnosis in such a case is crucial in that clinical outcomes are dependent on correct identification of the causative pathogen and timely administration of the proper antimicrobial treatment.

To the best of our knowledge, this is the first reported case of *A. kilianii* infection in the Asian Pacific region. Previously, this strain was reported only in European countries [3]. Because *A*. *kilianii* is a relatively newly identified strain, it was not identified by conventional biochemical identification and MALDI-TOF. All species of *Aggregatibacter* can be distinguished from *Haemophilus* by negative indole, urease, and ornithine decarboxylase tests, and the *A*. *kilianii* sp. is differentiated from three other *Aggregatibacter* species by a positive alanine-phenylalanine-proline arylamidase test. Because only a few cases of *A*. *kilianii* have been reported, its antibiotic susceptibility profile is unclear. Also, since a β-lactamase-producing strain of the HACEK group was reported, the results were very important in the treatment of this bacterium [7]. We were not able to make valid susceptibility interpretation because there was no susceptibiity criteria for E-test in the CLSI guideline. However, it was reported that the E-test MIC value was 98% consistent with the broth microdilution within 2 log dilution [8], we could carefully presumed that this strain was sensitive to the tested antibiotics.

The major limitation of this study is that it could not be verified that *A*. kilianii caused the infection or if it was a commensal. Because it was co-cultured with *S*. *constellatus*, and there are few reports on the clinical significance of *A. kilianii*, we could not distinguish the true causative organism. However, this study is a rare clinical report of a novel strain that, to the best of our knowledge, has no previously reported antibiotic profiles. Therefore, the provision of information on antimicrobial susceptibility is crucial.

In conclusion, this is the first reported case of *A. kilianii* infection outside Europe. This report highlights the importance of proper molecular identification for timely diagnosis and treatment of disease. It also provides information on the antimicrobial susceptibility of the organism for future reference in persons suspected of infection with *A*. *kilianii.*

## Data Availability

The 16 S rRNA sequences of *Aggregatibacter kilianii* are available in the GenBank database (Accession Number: ON421855 ).

## Abbreviations

CLSI: Clinical and Laboratory Standards Institute
CRP: C-reactive protein
ERBD: Endoscopic retrograde biliary drainage
MALDI-TOF: Matrix-assisted laser desorption/ionization time of-flight
NH: Neisseria-haemophilus
RNA: Ribose nucleic acid
